# Perception of community pharmacists about the work process of drug dispensing: a cross-sectional survey study

**DOI:** 10.1186/s12913-022-07528-y

**Published:** 2022-02-08

**Authors:** Sabrina Cerqueira Santos, Kérilin Stancine Santos Rocha, Dyego Carlos Souza Anacleto de Araújo, Elindayane Vieira de Souza, Lara Joana Santos Caxico Vieira, Sylmara Nayara Pereira dos Santos, Divaldo Pereira de Lyra Júnior

**Affiliations:** 1 Graduate Program in Pharmaceutical Sciences, Federal University of Sergipe, Avenue Marechal Rondon, Jd. Rosa Elze, São Cristóvão, Sergipe State Zip code 49100-000 Brazil; 2 Laboratory of Teaching and Research in Social Pharmacy (LEPFS), Department of Pharmacy, Federal University of Sergipe, Avenue Marechal Rondon, Jd. Rosa Elze, São Cristóvão, Sergipe Zip code 49100-000 Brazil

**Keywords:** Community pharmacy services, Pharmaceutical services, Pharmacies, Drug dispensing. Survey

## Abstract

**Background:**

Drug dispensing aims to promote rational medicine use. However, in many countries, the work processes are still not well defined. In this sense, the perception of pharmacists about dispensing practices presents an overview of how the service is being performed in the country and its main challenges. Thus, the purpose of this study was to determine the self-reported work process of Brazilian community pharmacists in relation to drug dispensing, challenges, and strategies for carrying out the service.

**Method:**

A cross-sectional survey was conducted between May and July 2021, with community pharmacists from all regions of Brazil. Pharmacists were invited to answer a validated, self-administered questionnaire, implemented through Google Forms, containing 33 questions related to the steps of drug dispensing (questions and counseling) and the main challenges and strategies to perform the service. The data were exported to Microsoft Office Excel and SPSS®. Multiple linear regression analysis was used to assess the association between responses and demographic information, with a significance level of less than 5% (*p* < 0.05). This study was approved by the Research Ethics Committee (number: 4.295.171).

**Results:**

A total of 625 community pharmacists responded to the survey. Most pharmacists reported always or frequently performing 17 (54%) of the 31 steps described in the instrument. The steps that pharmacists reported performing more frequently were forming the medication name (*n* = 569, 91.04%), verifying the completeness and adequacy of the prescription according to current legislation (*n* = 567, 90.72%) and providing counseling on dosage (*n* = 549, 87.84%). Documentation was the main step in which pharmacists reported never or rarely performing (*n* = 424, 67.84%). The results showed that there was a significant influence of the variables of public education institution, age, and postgraduate education on the frequency of dispensing steps (F(3, 621) = 14.884, *p* < 0.001; *R*^2^_ajdusted_ = 0,063).

**Conclusion:**

This study showed that most pharmacists reported always or frequently asking most of the questions and performing counseling contained in the instrument during drug dispensing. These results can contribute to an understanding of current dispensing practices and generate insights for developing strategies to qualify the service.

**Supplementary Information:**

The online version contains supplementary material available at 10.1186/s12913-022-07528-y.

## Background

Worldwide, community pharmacies are considered the main health establishments that provide access to medicines for the population. In the United States, in 2019, approximately 4.38 billion prescriptions were dispensed [1]. In Brazil, this number may be even higher, since this country is one of the five largest medicine consumers in the world [2]. Furthermore, there are more than 85 thousand community pharmacies in the country, which is considered the workplace of almost 80% of Brazilian pharmacists [3, 4].

In Brazil, people have access to medicines through private community pharmacies and/or pharmacies belonging to the Brazilian Health System (SUS), free of charge. Nationally regulated licensure is needed to open and operate a community pharmacy in Brazil. Although pharmacy ownership is not restricted to pharmacists, a legally responsible pharmacist should be employed and be ever-present at the pharmacy during opening hours. There are no rules regarding the location of pharmacies in Brazil in relation to demographic and geographical criteria [5, 6].

In this context, community pharmacists are the health professionals most accessible to the public [7]. During the coronavirus (SARS-COV2) pandemic, pharmacists screened patients with COVID-19, advising the population about the disease, the use of medications, and made referrals when appropriate [8, 9]. Among the services that pharmacists can perform in community pharmacies, drug dispensing is a clinical pharmacy service that ensures the provision of medicines and other health products through the analysis of technical and legal aspects of prescription, assessment of individual health needs, and performance of interventions in the medicine use process, which includes pharmaceutical counseling and documentation of interventions made [10–12].

Drug dispensing, when performed properly, promotes the rational use of medicines, providing effective and safe treatments, and improving the patient’s quality of life [13, 14]. However, although it is a service traditionally performed by pharmacists, there are still difficulties in understanding the drug dispensing by pharmacists, and other professionals, and the work processes of this service are not well defined [15, 16]. Studies performed in low-to middle-income countries, including Brazil, show that drug dispensing is often performed with insufficient counseling to patients and most of the time there is no record of the patient’s information and the interventions performed [17, 18]. As a result, the quality of care provided can be affected, compromising the medication use process.

In Brazil, self-reported studies have assessed pharmacists’ knowledge of drug dispensing, focusing on regulatory aspects and studies that generally concern activities carried out by community pharmacists [3, 19–21]. However, studies on drug dispensing practices at the national level are still scarce and can present an overview of how the service is being carried out in the country and its main challenges. Thus, the objective of this study was to determine the self-reported work process of Brazilian community pharmacists in relation to drug dispensing, the challenges, and strategies for carrying out the service.

## Methods

### Study design

A cross-sectional survey was conducted in Brazil, between May and July 2021, using the recommendations proposed by Kelley (2003) [22], Bennett et al. (2011) [23], and Rybakov et al. (2020) [24]. All methods were carried out in accordance with relevant guidelines and regulations.

### Questionnaire development

The questionnaire used in this study was developed by two researchers (KSSR; SCC), through meetings, based on instruments validated by Rocha et al. (2020) [25] and Cerqueira-Santos et al. (2019) [26]. The content validity of the questionnaire was performed using the Delphi technique. Thus, a panel of six experts in drug dispensing from Brazil, including three researchers and three pharmacists were invited to participate in Delphi, following literature recommendations [27]. The experts were selected based on the adaptation of Fehring’s scoring model. A minimum score of five points was required to warrant selection [28].

In the first round of the Delphi, the experts were informed about the study’s aims and the instructions necessary to carry out the content validity assessment. An electronic assessment form was sent via email to the experts. They were asked to rate each item using the following criteria: simplicity (the content of the item expresses a single idea), language clarity (i.e., the language is clear, understandable, and appropriate for the target population), and practical pertinence (i.e., the item assesses a concept that is of interest to the target population), using a 5-point Likert scale. The experts were also invited to provide additional comments about each questionnaire item. The content validity coefficient (CVC) was calculated to quantify content validity. The cutoff score was used to indicate a satisfactory level of simplicity, language clarity, and practical pertinence was ≥0.80 (for each item and the entire scale) [29]. The deadline for the evaluation and judgment of the items was 30 days, with weekly reminders sent via e-mail.

During the second round, the expert panel had access to all the results obtained during the first round. The expert panel was instructed to analyze changes in items and evaluate them according to the criteria (simplicity, language clarity, and practical pertinence), using the 5-point Likert scale. The content validity coefficient was calculated to quantify the content validity. The cutoff score used was ≥0.80 (for each item and the entire scale). The deadline for evaluation and judgment of items was 30 days, and weekly reminders were sent to the expert panel members by e-mail. After content validity, a pilot test was carried out to evaluate the clarity, comprehension, and time of completion of the questionnaire with 10 purposely selected community pharmacists who were excluded from the final survey.

### Survey application

#### Sample selection/participants

A convenience sample and snowball strategy were used to select participants [22]. The study included pharmacists who work in community pharmacies, performed drug dispensing in their routine practice, of both sexes, and who agreed to voluntarily participate in the research. In this study, there were no exclusion criteria.

#### Data collection

The anonymous, self-administered questionnaire was sent to pharmacists in all regions of Brazil through an online platform (Google Forms) between May and July 2021. The invitation to pharmacists to participate in the research was advertised through social media and in partnership with Brazilian Regional Pharmacy Council through email and websites. Upon accessing the online platform, only community pharmacists were instructed to respond to the survey. All questions in the questionnaire were marked as mandatory to avoid incomplete answers. No financial incentives were provided to the participants.

#### Sample size calculation

Considering a population of 221.258 pharmacists registered with the Regional Pharmacy Councils in 2020 in Brazil [4], a confidence level of 95% and a sampling error of 5%, a minimum of 384 pharmacists were required for this study.

#### Data analysis

The data were presented using descriptive statistics (mean, standard deviation, and percentage). We assumed a normal distribution of data based on skewness (−.223) and kurtosis (−.147) measures. A multiple linear regression analysis (forward method) was performed to investigate the variables: sex, age, time since graduation, graduate institution, postgraduate education, type of postgraduate degree (stricto sensu or lato sensu), experience with drug dispensing, workplace, and the frequency of the drug dispensing steps. It is worth noting that the the stricto sensu postgraduate course refers to the master’s and PhD programs. The lato sensu postgraduate programs comprise specialization programs with a minimum workload of 360 h, including, for example, MBA (Master Business Administration) and residency program. For the statistical analysis, a total score was established for the frequency of steps performed by the pharmacist during drug dispensing, with 1 point representing “never,” 2 “rarely,” 3 “sometimes,” 4 “often” and 5 “ever.” The minimum score was 31 and the maximum score was 155 points. The data from the online survey were exported into Microsoft Office Excel and SPSS® (version 22.0). Missing data were handled using a single imputation [30].

## Results

### Survey instrument development

Of the six experts invited to compose the panel of experts, six returned their assessments (100% rate of return). Half of the experts were between 31 and 40 years of age (*n* = 3). Their mean professional experience with drug dispensing was 8.33 ± 4.03 years. Half of the experts had a PhD (*n* = 3, 50%) and specialized in a clinical area (*n* = 3, 50%), such as Clinical Paharmacy and Pharmaceutical Care. In first round of the Delphi, with all the three criteria, the questionnaire yielded coefficients ≥0.80 (range = 0.87–1.0). Only one item obtained a coefficient of 0.70 for the clarity criterion and has been resubmitted for review. Although most items achieved a CVC ≥ 0.80, some suggestions were relevant and resulted in item changes. The main suggestions were related to changes in the writing of some items and the division of some questions. Of the six experts who participated in the first round, five completed the second round (83, 33% rate of return). For all three criteria, the questionnaire yielded coefficients that were ≥ 0.80 (range = 0.85–1.0), thus generating the final version of the questionnaire.

The questionnaire was divided into the following sections: i) pharmacists’ perceptions about the work process of drug dispensing (31 items), ii) strategies and challenges for performing drug dispensing (2 items), and iii) sociodemographic data. The items in the first section refers to the steps (questions and counseling) that can be performed during drug dispensing. The survey items were answered using a 5-point Likert scale ranging from never to always and multiple-choice questions. When appropriate, an additional free text area was provided (Additional file 1).

### Survey application

A total of 625 community pharmacists responded to the survey. It was not possible to calculate the response rate because of the survey’s multiple means of dissemination. Of this sample, 72.96% (*n* = 456) were female, the mean age of pharmacists was 33 ± 7.44 years, and the average time since pharmacy graduation was 6.22 ± 6.21 years. Of the 273 pharmacists who completed a postgraduate course in the clinical area, 254 (91.69%) had done a lato sensu postgraduate course. The characteristics of these professionals are presented in Table 1.

**Table 1:** Characteristics of pharmacists who responded the survey (*n* = 625)

Characteristics	Pharmacists (%)
Sex	
Female	456 (72.96%)
Male	169 (27.04%)
Age	
21–30 years	286 (45.76%)
31–40 years	252 (40.32%)
More than 40 years	87 (13.92%)
Time since graduation	
Up to1 year	84 (13.44%)
From 2 to 4 years	256 (40.96%)
From 5 to 7 years	112 (17.92%)
More than 7 years	173 (27.68%)
Graduate institution	
Public	215 (34.40%)
Private	410 (65.60%)
Workplace^a^	
Private community pharmacy chain	422 (67.52%)
Independent community pharmacy	149 (23.84%)
Public pharmacy	102 (16.32%)
Postgraduate in the clinical area	
Yes	273 (43.68%)
No	352 (56.32%)
Time that works with dispensing (years)	
Up to1 year	157 (25.12%)
From 2 to 4 years	229 (36.64%)
From 5 to 7 years	85 (13.60%)
More than 7 years	154 (24.64%)
Brazil region	
Northeast	359 (57.44%)
Southeast	112 (17.92%)
South	74 (11.84%)
Midwest	67 (10.72%)
North	13 (2.08%)

^a^The pharmacist can work in more than one location

The frequency with which pharmacists reported performing some steps of the drug dispensing service is described in Table 2. Most professionals reported always or frequently performing 17 (54.00%) of the 31 steps described in the instrument. The average score for the frequency of steps performed by the pharmacist during dispensing was 112 ± 20 points (54–155). Item 7 (inform the medicine name) was the item that pharmacists reported performing most frequently (always or frequently) (*n* = 569, 91.04%), followed by item 1 (verifying the completeness and adequacy of the prescription according to current legislation) (*n* = 567, 90.72%), item 14 (counsel on dosage) (*n* = 549, 87.84%), item 2 (verifies whether the drug dispensing is for the patient, caregiver, or other person) (*n* = 534, 85.44%), and finally item 12 (counsel on how to use the pharmaceutical form of the medicine) (*n* = 497, 79.752%).

**Table 2:** Frequency with which the steps are performed by pharmacists during drug dispensing (*n* = 625)

Steps	Frequency with which the pharmacist performs step n (%)				
	Always	Frequently	Sometimes	Rarely	Never
1.Verify the completeness and adequacy of the prescription according to current legislation	412 (65.92%) *	155 (24.80%)	45 (7.20%)	11 (1.76%)	2 (0.32%)
2.Verify if the dispensing is for the patient, caregiver, or other person	308 (49.28%) *	226 (36.16%) *	75 (12.00%)	12 (1.92%)	4 (0.64%)
3. Verify if this is the first time the patient uses the medicine	157 (25.12%)	253 (40.48%) *	173 (27.68%)	36 (5.76%)	6 (0.96%)
4. Verify if there are contraindications for the use of the medicine (e.g., allergy, pregnancy, health conditions, among others)	129 (20.54%)	210 (33.60%)	200 (32.00%)	76 (12.16%)	10 (1.60%)
5.Verify if the patient uses other medications or has other health problems	93 (14.88%)	186 (29.76%)	231 (36.96%) *	102 (16.32%)	13 (2.08%)
6. Verify if the patient is familiar with the indication(s) of the medication	126 (20.16%)	220 (35.20%)*	202 (32.32%)	73 (11.68%)	4 (0.64%)
7. Inform the medication name	431 (68.96%) *	138 (22.08%)	40 (6.40%)	11 (1.76%)	5 (0.80%)
8. Counsel on the clinical condition (disease/sign/symptom) for which the medication was prescribed	125 (20.00%)	257 (41.12%) *	182 (29.12%)	53 (8.48%)	8 (1.28%)
9. Counsel about medication indication(s)	196 (31.36%)	243 (38.88%) *	153 (24.48%)	28 (4.48%)	5 (0.80%)
10. Counsel on therapeutic goals	75 (12.00%)	169 (27.04%)	214 (34.24%)	127 (20.32%)	40 (6.40%)
11. Verify if the patient knows about correct use of the medication	193 (30.88%)	218 (34.88%)	163 (26.08%)	43 (6.88%)	8 (1.28%)
12. Counsel on how to use the pharmaceutical form of the medication	281 (44.96%) *	216 (34.56%)	106 (16.96%)	20 (3.20%)	2 (0.32%)
13. Inform the route of administration of the medication	310 (49.60%) *	177 (28.32%)	97 (15.52%)	36 (5.76%)	5 (0.80%)
14. Counsel on dosage (dose, frequency, and duration of treatment)	338 (54.08%) *	211 (33.76%)	61 (9.76%)	14 (2.24%)	1 (0.16%)
15. Counsel about the time to take the medication	296 (47.36%) *	204 (32.64%)	103 (16.48%)	18 (2.88%)	4 (0.64%)
16. Counsel on time for the medication to take effect	93 (14.88%)	140 (22.40%)	223 (35.68%) *	148 (23.68%)	21 (3.36%)
17. Counsel on interactions (drug/drug; drug/food; drug/alcohol)	79 (12.64%)	159 (25.44%)	227 (36.32%) *	136 (21.76%)	24 (3.84%)
18. Counsel on how to monitor the health problem	66 (10.56%)	178 (28.48%)	215 (34.40%)	139 (22.24%)	27 (4.32%)
19. Counsel on non-pharmacological treatment	82 (13.12%)	178 (28.48%)	239 (38.24%) *	96 (15.36%)	30 (4.80%)
20. Counsel on medication storage	126 (20.16%)	170 (27.20%)	186 (29.76%)	115 (18.40%)	28 (4.48%)
21. Counsel on medication disposal	71 (11.36%)	105 (16.80%)	175 (28.00%)	199 (31.84%)	75 (12.00%)
22. Verify if the patient knows about aspects related to the treatment safety (e.g., have you ever felt something different when using this medication?)	61 (9.76%)	110 (17.60%)	245 (39.20%) *	158 (25.28%)	51 (8.16%)
23. Counsel on precautions regarding the medication use (e.g., for medications that cause drowsiness, pay extra attention when driving or operating machinery)	184 (29.44%)	221 (35.36%) *	148 (23.68%)	53 (8.48%)	19 (3.04%)
24. Counsel on adverse drug reactions	79 (12.64%)	161 (25.76%)	276 (44.16%) *	91 (14.56%)	18 (2.88%)
25. Counsel on the consequences of using the medication in the long-term, when applicable	129 (20.64%)	172 (27.52%)	212 (33.92%)	88 (14.08%)	24 (3.84%)
26. Verify if the patient is aware of aspects related to medication adherence (e.g.: Some people forget to take their medication, does this happen to you? What are your concerns regarding the use of this medication?)	92 (14.72%)	185 (29.60%)	204 (32.64%)	112 (17.92%)	32 (5.12%)
27. Counsel on management in case of a missed dose	81 (12.96%)	145 (23.20%)	217 (34.72%)	141 (22.56%)	41 (6.56%)
28. Counsel on the importance of correct use of medicine and medication adherence	215 (34.40%)	242 (38.72%) *	121 (19.36%)	38 (6.08%)	9 (1.44%)
29. Confirm the patient’s understanding of the counseling provided in the drug dispensing	199 (31.84%)	238 (38.08%) *	122 (19.52%)	52 (8.32%)	14 (2.24%)
30. Documents the interventions performed in the drug dispensing	66 (10.56%)	52 (8.32%)	83 (13.28%)	137 (21.92%) *	287 (45.92%) *
31. Refers the patient to other health professionals and/or clinical pharmacy services, when necessary	185 (29.6%)	162 (25.92%)	137 (21.92%)	94 (15.04%)	47 (7.52%)

*Most frequently reported steps for each Likert scale level

The dispensing steps that pharmacists reported to be rarely or never performing were related to item 30 (Documents the interventions performed in the drug dispensing) (*n* = 424, 67.84%), followed by item 21 (counsel on medicine disposal) (*n* = 274, 43.84%) and 22 (Verify if the patient knows about aspects related to the treatment safety) (*n* = 209, 33.44%) and 16 (counsel on time for the medicine to take effect) (*n* = 169, 27.04%).

The results showed that there is a significant influence of the predictor variables of public graduate institution, increasing age and postgraduate on the frequency scores of the dispensing steps (F(3.621) = 14.884, *p* < 0.001; R2adjusted = 0.063). Table 3 presents the coefficients for all significant predictor variables. The variable that most strongly impacted the scores was public graduate institution, explaining 4.6% of the outcome. The other variables were related to only 1.9% of the score variance. The variables time since graduation, workplace, type of postgraduate degree, years of experience with dispensing and sex had no significant impact (*p* < 0.05) (Table 4).

**Table 3:** Predictive variables of frequency scores of dispensing steps

Variables	Standardized coefficients	*t*	Sig.	*R*^*2*^	Δ*R*^*2*^
	*Beta*				
(Constant)	–	25.792	0.000	–	–
Public graduate institution	0.213	5.491	0.000	0.046	–
Age	0.101	2.595	0.010	0.057	0.012
Postgraduate	0.086	2.213	0.027	0.063	0.007

**Table 4:** Predictor variables excluded from the model

Predictors	*Beta*	*t*	Sig.
Time since graduation (years)	−0.086	−1.619	0.106
Work in private community pharmacy chain	−0.001	−0.030	0.976
Work in independent community pharmacy	0.021	0.544	0.587
Postgraduate Stricto Sensu	−0.021	−0.515	0.607
Time that works with dispensing (years)	0.066	1.451	0.147
Sex (Female)	0.070	1.787	0.074

Pharmacists used different strategies for patient counseling during drug dispensing and most of them reported using more than one strategy (*n* = 528, 84.48%) (Table 5). The main strategy was verbal information (*n* = 611, 97.76%), followed by written information (*n* = 412, 65.92%). The main challenges in performing drug dispensing are described in Table 6, with the most reported being the large number of patients using the community pharmacy (*n* = 494, 79.04%), refusal of counseling by the patient because they had already been counseled by the physician (*n* = 314, 50.24%), and the view of the pharmacy as a commercial establishment (*n* = 312, 49.92%). Other challenges reported were lack of patient time, too many activities to be performed, illegible prescriptions, and limited views of managers and patients regarding the role of pharmacists. Most pharmacists also reported more than one challenge (*n* = 550, 88.00%), and 24 professionals (3.84%) stated that there were no impediments to patient counseling.

**Table 5:** Strategies used to patient counseling during drug dispensing (*n* = 625)

Strategies for pharmacist’s counseling during drug dispensing	Pharmacists (%)
Verbal information	611 (97.76%)
Written information	412 (65.92%)
Practical demonstration of correct drug handling	358 (57.28%)
Provision of pictograms (image or symbol representing a word or phrase) and/or other illustrations	143 (22.88%)
Provision of a timetable for organize a medication schedule	183 (29.28%)
Information material (e.g., pamphlet, flyer, among others)	66 (10.56%)
Diary for self-monitoring (diary for the patient to record symptoms, clinical parameters)	37 (5.92%)

**Table 6:** Main challenges to perform drug dispensing (*n* = 625)

Challenges to perform drug dispensing	Pharmacists (%)
Large number of patients using the community pharmacy	494 (79.04%)
View of the pharmacy as a commercial establishment	312 (49.92%)
Provision of more than one medicine during dispensing	205 (32.80%)
Absence of a semi-private place to carry out the drug dispensing	276 (44.16%)
Limitation of knowledge and clinical skills for perform drug dispensing	88 (14.08%)
Refusal of counseling by the patient	229 (36.64%)
No request for counseling by the patient	283 (45.28%)
Refusal of counsel by the patient because he had already been counseled by the physician	314 (50.24%)

## Discussion

The present study documented the self-reported work process of community pharmacists in relation to drug dispensing, strategies, and challenges in performing the service. Most pharmacists reported always or frequently performing most steps (questions and counseling) during dispensing. Similar to the results of this work, studies show that the main pharmaceutical counseling provided to the patient during drug dispensing is about dosage and use of the medicine [15, 31, 32]. Despite this, the literature has shown low rates of pharmaceutical counseling during drug dispensing, often restricting this service to drug delivery [33–35]. It is important to highlight that drug dispensing, as a clinical pharmacy service, requires pharmacists to identify the needs of each patient by asking appropriate questions, and as a result, provide the necessary counseling for rational medicine use [25, 36]. Therefore, understanding the process and the main challenges are essential to improve the service, enabling the design of specific strategies for each reality.

The results of this study showed a relationship between the graduate institution and the frequency with which questions were asked, and counseling was given according to pharmacists’ reports. This difference had a small size effect, but studies have shown differences in pharmacy undergraduate institutions. In Brazil, there are approximately 429 pharmacy degree courses in private institutions and 73 courses public institutions, more than four times the number of degree courses in the United States [37]. Despite efforts to improve the clinical training of Brazilian pharmacists, such as the publication of the new Guidelines for the Undergraduate Course in Pharmacy that recommend that 50% of the workload of the course is in the healthcare lines, the unreasonable number of higher education institutions, different curricular structures, and teaching methods may have influenced the work process of drug dispensing [37–40].

Still on the subject of pharmacy undergraduate institutions, indicators related to student performance, faculty, and conditions offered for the development of the learning process showed that the quality of public institutions in Brazil is better than that of private institutions [41]. In contrast, countries like the United States, England, and Australia have accredited institutions that harmonize pharmacy education, providing higher quality and uniformity in higher education [42–44]. Thus, it is expected that Brazilian institutions, public or private, focus to the needs of society, in order to develop critical and competent professionals for the provision of clinical pharmacy services, such as drug dispensing.

Regarding postgraduate education in the clinical area, a significant difference was also observed in the frequency of questions and counseling during drug dispensing. Patient-centered care requires pharmacists to be involved in the continuous improvement of knowledge, skills, and performance [45, 46]. According to the literature, some barriers prevent pharmacists from engaging in continuing education activities, such as the lack of specific policies, time, and motivation [45, 47, 48]. Some countries, such as the United States, have legislation mandating pharmaceutical continuing education for pharmacists’ relicensure, which involves, for example, specification of a certain number of live and home study credit hours, and specific topic requirements [49]. In contrast, in Brazil, there is no requirement for annual accreditation hours for professional relicensure. Therefore, Brazilian institutions should be inspired by the example set by other countries to overcome these challenges, as well as to make pharmacists aware of the need for continuous improvement in the provision of patient care.

In this study, it was observed that the older the professionals, the more they reported questions asked and provided counseling to patients. According to the literature, counseling skills on medicine use can improve with age, and professionals can feel more confident in providing clinical pharmacy services in the health field [46, 50–52]. It is important to highlight that other factors were not evaluated in the statistical model of this study and can influence the counseling rates during drug dispensing, such as the complexity of the clinical case, lack of time, large number of patients using the community pharmacy, pharmacists’ knowledge, and patients’ attitudes, among others [53–55]. Therefore, it is necessary to be careful when analyzing the results, as age is not the only, or the most important, factor that can influence the process of drug dispensing.

Most pharmacists in this study reported not documenting drug dispensing. Documentation is an essential component of the care process, as it facilitates communication and enables the measurement of results [56, 57]. In high-income countries, the documentation of interventions during drug dispensing is well consolidated, but there are factors that hinder this process in low- and middle-income countries, such as the reduced number of professionals in the pharmacy, lack of time, and little understanding of the importance of this component of practice [56, 58]. Thus, teaching-learning strategies must be incorporated in both undergraduate and continuing education to develop, in students and professionals, the skills needed to overcome this main challenge in patient care [26]. In addition, changes in organizational culture are needed to encourage professionals to document their practices. The use of technologies such as big data and applications can facilitate the incorporation of drug dispensing documentation in Brazilian community pharmacies [59, 60].

In this study, the main challenges in dispensing reported by pharmacists were related to the large number of patients using the community pharmacy and the patient’s behavior. Similar results have been described in the literature, and the most common barriers in providing patient counseling were patient factors, the large number of patients using the community pharmacy, and lack of professional hours available [15, 32, 61]. In addition, peak hours are associated with patient dissatisfaction, which can negatively affect their experience [62]. As many patients still do not understand the role of pharmacists in improving health outcomes, strategies such as the organization of drug dispensing work processes, the professional’s proactive attitude, and the use of technologies can facilitate patient care during dispensing [63, 64].

This study had several strengths and limitations. The main limitation is that self-reported research may have generated socially desirable responses and did not reliably represent reality. Furthermore, as the representativeness of the sample could not be verified caution should be exercised in generalising these findings. To our knowledge, this is the first study carried out in different regions of a continental country such as Brazil that sought to understand the self-reported work process of Brazilian community pharmacists in relation to drug dispensing and the challenges and strategies for carrying out the service.

## Conclusion

Most study participants reported performing most of the necessary questions and counseling during drug dispensing. The frequency of questions and counseling was influenced by factors such as the age of the pharmacist, the educational institution, and continuing education, such as postgraduate education. The present study observed problems existing in Brazilian drug dispensing practices, such as the large number of patients using the community pharmacy, refusal of counseling by the patient, and the view of the pharmacy as a commercial establishment, among others. The main strategies for pharmacists’ counseling during drug dispensing were verbal and written information. The data obtained can contribute to understanding the current reality and generating insights for community pharmacists, researchers, health policy makers, and other stakeholders to optimize the work process of drug dispensing and its impact on patient outcomes.

## Supplementary Information

**Additional file 1.**

## Data Availability

The datasets used and/or analysed during the current study are available from the corresponding author on reasonable request.

## Abbreviation

CVC: Content validity coefficient
