# Supplementary material for: Perception of community pharmacists about the work process of drug dispensing: a cross-sectional survey study
Source: BMC Health Serv Res. 2022 Feb 8;22:161. doi: 10.1186/s12913-022-07528-y (PMC8822789; doi:10.1186/s12913-022-07528-y)
Supplement: Supplementary file 1 — Additional file 1. [file 12913_2022_7528_MOESM1_ESM.pdf]

QUESTIONNAIRE: " WORK PROCESS OF DRUG DISPENSING IN COMMUNITY PHARMACIES"

Drug dispensing		
**How often do you take the following steps in drug dispensing?**		
1.	Verify the completeness and adequacy of the prescription according to current legislation	(1) Never (2) Rarely (3) Sometimes (4) Frequently (5) Always
2.	Verify if the dispensing is for the patient, caregiver, or other person	(1) Never (2) Rarely (3) Sometimes (4) Frequently (5) Always
3.	Verify if this is the first time the patient uses the medicine	(1) Never (2) Rarely (3) Sometimes (4) Frequently (5) Always
4.	Verify if there are contraindications for the use of the medicine (e.g., allergy, pregnancy, health conditions, among others)	(1) Never (2) Rarely (3) Sometimes (4) Frequently (5) Always
5.	Verify if the patient uses other medications or has other health problems	(1) Never (2) Rarely (3) Sometimes (4) Frequently (5) Always
6.	Verify if the patient is familiar with the indication(s) of the medication	(1) Never (2) Rarely (3) Sometimes (4) Frequently (5) Always
7.	Inform the medication name	(1) Never (2) Rarely (3) Sometimes (4) Frequently (5) Always
8.	Counsel on the clinical condition (disease/sign/symptom) for which the medication was prescribed	(1) Never (2) Rarely (3) Sometimes (4) Frequently (5) Always
9.	Counsel about medication indication(s)	(1) Never (2) Rarely (3) Sometimes (4) Frequently (5) Always
10.	Counsel on therapeutic goals	(1) Never (2) Rarely (3) Sometimes (4) Frequently (5) Always
11.	Verify if the patient knows about correct use of the medication	(1) Never (2) Rarely (3) Sometimes (4) Frequently (5) Always
12.	Counsel on how to use the pharmaceutical form of the medication	(1) Never (2) Rarely (3) Sometimes (4) Frequently (5) Always
13.	Inform the route of administration of the medication	(1) Never (2) Rarely (3) Sometimes (4) Frequently (5) Always
14.	Counsel on dosage (dose, frequency, and duration of treatment)	(1) Never (2) Rarely (3) Sometimes (4) Frequently (5) Always
15.	Counsel about the time to take the medication	(1) Never (2) Rarely (3) Sometimes (4) Frequently (5) Always
16.	Counsel on time for the medication to take effect	(1) Never (2) Rarely (3) Sometimes (4) Frequently (5) Always
17.	Counsel on interactions (drug/drug; drug/food; drug/alcohol)	(1) Never (2) Rarely (3) Sometimes (4) Frequently (5) Always
18.	Counsel on how to monitor the health problem	(1) Never (2) Rarely (3) Sometimes (4) Frequently (5) Always
19.	Counsel on non-pharmacological treatment	(1) Never (2) Rarely (3) Sometimes (4) Frequently (5) Always
20.	Counsel on medication storage	(1) Never (2) Rarely (3) Sometimes (4) Frequently (5) Always

21.	Counsel on medication disposal	(1) Never (2) Rarely (3) Sometimes (4) Frequently (5) Always
22.	Verify if the patient knows about aspects related to the treatment safety (e.g., have you ever felt something different when using this medication?)	(1) Never (2) Rarely (3) Sometimes (4) Frequently (5) Always
23.	Counsel on precautions regarding the medication use (e.g., for medications that cause drowsiness, pay extra attention when driving or operating machinery)	(1) Never (2) Rarely (3) Sometimes (4) Frequently (5) Always
24.	Counsel on adverse drug reactions	(1) Never (2) Rarely (3) Sometimes (4) Frequently (5) Always
25.	Counsel on the consequences of using the medication in the long-term, when applicable	(1) Never (2) Rarely (3) Sometimes (4) Frequently (5) Always
26.	Verify if the patient is aware of aspects related to medication adherence (e.g.: Some people forget to take their medication, does this happen to you? What are your concerns regarding the use of this medication?)	(1) Never (2) Rarely (3) Sometimes (4) Frequently (5) Always
27.	Counsel on management in case of a missed dose	(1) Never (2) Rarely (3) Sometimes (4) Frequently (5) Always
28.	Counsel on the importance of correct use of medicine and medication adherence	(1) Never (2) Rarely (3) Sometimes (4) Frequently (5) Always
29.	Confirm the patient's understanding of the counseling provided in the drug dispensing	(1) Never (2) Rarely (3) Sometimes (4) Frequently (5) Always
30.	Documents the interventions performed in the drug dispensing	(1) Never (2) Rarely (3) Sometimes (4) Frequently (5) Always
31.	Refers the patient to other health professionals and/or clinical pharmacy services, when necessary	(1) Never (2) Rarely (3) Sometimes (4) Frequently (5) Always

32. What strategies are used to patient counseling during drug dispensing?

( ) Verbal information

( ) Written information

( ) Practical demonstration of correct drug handling

( ) Provision of pictograms (image or symbol representing a word or phrase) and/or other illustrations

( ) Provision of a timetable for organize a medication schedule

( ) Information material (e.g., pamphlet, flyer, among others)

( ) Diary for self-monitoring (diary for the patient to record symptoms, clinical parameters)

( ) Others. Which?

33. What are the main challenges to perform drug dispensing?

( ) Large number of patients using the community pharmacy

( ) View of the pharmacy as a commercial establishment

( ) Provision of more than one medicine during dispensing

( ) Absence of a semi-private place to carry out the drug dispensing

( ) Limitation of knowledge and clinical skills for perform drug dispensing

( ) Refusal of counseling by the patient

( ) No request for counseling by the patient

( ) Refusal of counsel by the patient because he had already been counseled by the physician

( ) Without limitations, patient counseling is always carried out during drug dispensing

( ) Others. Which?
